# Developing a utility index for the Aberrant Behavior Checklist (ABC-C) for fragile X syndrome

**DOI:** 10.1007/s11136-014-0759-8

**Published:** 2014-07-26

**Authors:** Cicely Kerr, Katie Breheny, Andrew Lloyd, John Brazier, Donald B. Bailey, Elizabeth Berry-Kravis, Jonathan Cohen, Jennifer Petrillo

**Affiliations:** 1 ICON Patient Reported Outcomes, Seacourt Tower, West Way, Oxford, OX2 0JJ UK; 2 Present Address: University of Birmingham, Birmingham, UK; 3 School of Health and Related Research (ScHARR), The University of Sheffield, Sheffield, UK; 4 RTI International, Research Triangle Park, NC USA; 5 Departments of Pediatrics, Neurological Sciences, and Biochemistry, Rush University Medical Center, Chicago, IL USA; 6 Fragile X Alliance Inc, Melbourne, Australia; 7 Centre for Developmental Disability Health Victoria, Monash University, Melbourne, Australia; 8 Novartis AG, Basel, Switzerland

**Keywords:** Fragile X syndrome, Aberrant Behavior Checklist, TTO, Lead-time, Utility index

## Abstract

**Purpose:**

This study aimed to develop a utility index (the ABC-UI) from the Aberrant Behavior Checklist-Community (ABC-C), for use in quantifying the benefit of emerging treatments for fragile X syndrome (FXS).

**Methods:**

The ABC-C is a proxy-completed assessment of behaviour and is a widely used measure in FXS. A subset of ABC-C items across seven dimensions was identified to include in health state descriptions. This item reduction process was based on item performance, factor analysis and Rasch analysis performed on an observational study dataset, and consultation with five clinical experts and a methodological expert. Dimensions were combined into health states using an orthogonal design and valued using time trade-off (TTO), with lead-time TTO methods used where TTO indicated a state valued as worse than dead. Preference weights were estimated using mean, individual level, ordinary least squares and random-effects maximum likelihood estimation [RE (MLE)] regression models.

**Results:**

A representative sample of the UK general public (*n* = 349; mean age 35.8 years, 58.2 % female) each valued 12 health states. Mean observed values ranged from 0.92 to 0.16 for best to worst health states. The RE (MLE) model performed best based on number of significant coefficients and mean absolute error of 0.018. Mean utilities predicted by the model covered a similar range to that observed.

**Conclusions:**

The ABC-UI estimates a wide range of utilities from patient-level FXS ABC-C data, allowing estimation of FXS health-related quality of life impact for economic evaluation from an established FXS clinical trial instrument.

## Introduction

Fragile X syndrome (FXS) is a genetic condition caused by a mutation in the *FMR1* gene resulting in cognitive impairment and behavioural problems [1]. FXS is the most common inherited form of intellectual disability, affecting approximately one in 4,000 males and one in 8,000 females [2]. Behavioural characteristics include anxiety, aggression, hyperarousal, attention deficits, hyperactivity, irritability, self-injurious and avoidant behaviour [3]. Males will typically have intellectual disabilities linked to below average IQ. Language deficits are common, as well as problems with sequential processing, working memory and attention [4]. Psychiatric problems such as generalised anxiety disorder, social phobia and obsessive compulsive disorder were found to occur in 83 % of individuals with FXS [5]. Approximately 50 % of males with FXS will also have an autistic spectrum disorder (ASD) [6]. FXS can exert a substantial burden on caregivers [7], and many patients are unable to live independently [8].

Recent research has resulted in a new understanding of the molecular pathways affected by FXS, and a new generation of targeted treatments is currently being tested in clinical trials [9, 10]. Given the prevalence of social and behavioural problems in FXS, one commonly used measure is the Aberrant Behavior Checklist-Community Edition (ABC-C), a proxy-completed instrument for rating maladaptive and inappropriate behaviours of individuals with intellectual disabilities [11]. It has been shown to be sensitive in FXS [12–15] and is commonly adopted as a primary outcome measure in clinical trials [16]. The 58 item ABC-C measures problem behaviour in five domains: hyperactivity, socially unresponsive/lethargic behaviour, stereotypy, inappropriate speech and irritability [11]. Recently, an adjusted factor structure for individuals with FXS has been reported [16], which identified a sixth ABC-C domain in FXS, which separates out social avoidance behaviour from socially unresponsive/lethargic behaviour (ABC for FXS).

While caregiver-rated scales can demonstrate the efficacy of an intervention on key characteristics of FXS, only limited data are available regarding the impact of FXS on health-related quality of life (HRQL), particularly for adults. Many decision makers such as the National Institute for Health and Care Excellence (NICE) in the UK prefer to evaluate treatments in terms of impact on survival and HRQL using the quality-adjusted life year (QALY) metric. The estimation of QALYs relies upon HRQL scales that reflect the value (or utility) that people place on health states on a scale from zero (dead) to one (full health). A lack of HRQL data and even suitable HRQL measures in FXS limits ability to estimate QALYs for this condition.

Different methods exist for capturing HRQL data suitable for estimating QALYs, the most common of which, and preferred by reimbursement agencies such as NICE [17], is use of standardised generic questionnaires where the patient describes their HRQL in a series of questions. Scoring/preference weights are applied to these responses to estimate a utility score. For the purposes of reimbursement review, commonly, it is the societal perspective that is important [18, 19], so the scoring weights are elicited from the general public in a separate exercise. Examples of such measures include the EQ-5D, SF-6D and Health Utilities Index (HUI) [20–23].

However, generic HRQL measures such as the EQ-5D (covering mobility, self-care, usual activities, pain/discomfort and anxiety/depression) may not accurately capture the impact of certain aspects of FXS, a condition with predominantly behavioural, social and cognitive characteristics. Also, the more subjective aspects of HRQL such as mood, affect, psychological state or pain can be difficult for a proxy to judge, as evidenced by higher rates of missing data on more subjective domains completed by parents of children with an ASD [24]. Agreement between patient and proxy assessments of HRQL has been found to depend on the concreteness, visibility and importance of aspects of HRQL [25].

An alternative approach is to develop a utility scoring algorithm, or index, from an existing disease-specific measure or one designed to measure problems associated with certain types of conditions. Disease-specific descriptive systems include items relevant to the patient’s condition that may not be captured by generic measures [26, 27]. Where a validated disease-/problem-specific measure is commonly used to capture primary outcome data in clinical trials, the ability to estimate utilities from these same data is an added benefit. As the ABC-C in its original form cannot be used to estimate QALYs, the current study was designed to develop and evaluate an ABC-utility index [ABC-UI] to report health state utility scores for children, adolescents and adults with FXS based on patient-level responses to the ABC-C.

## Methods

### Design

Methodological steps to derive a utility index for the ABC-C followed those described for other condition-specific preference-weighted scoring algorithms [28, 29] including reducing the number of items; selecting the best items for forming a health state classification using psychometric analysis; reducing the number levels on each item; valuing a sample of states defined by the health state classification; and modelling the health state data in order to generate a scoring algorithm.

### Development of health state descriptions

Selection of ABC-C items for health state development was based on a combination of statistical analysis and expert input. Secondary analysis of ABC-C data from a study involving 350 US FXS caregivers [30] was undertaken. Thirty-one out of 58 ABC-C items were dropped on the basis of preliminary statistical analysis using the following considerations: missing data >4 %, evidence of floor/ceiling effects, item variability (<10 % of responses on two or more adjacent scale points), range, inter-item correlation, evidence of cross-loading on two or more domains and poor factor loading (principle components analysis, varimax and promax explored). Item selection was further informed by Rasch analysis on the remaining items exploring item difficulty and differential item functioning (DIF) for age (children/adolescents vs. adults) and gender. Little variation in Rasch item difficulty was found in the dataset (ranging from 1.18 logit (ABC-C:44) to 2.06 logit (ABC-C:53)), providing insufficient guidance for selecting items covering a range of severity. Nineteen items showed significant DIF by age and 13 by gender (*p* < 0.05). Guided by the DIF results, items were selected for consistency in functioning across age and gender.

Five clinical experts (*n* = 5) with extensive collective experience of working with individuals with FXS in the US, UK and Australia (named as co-authors or in acknowledgements) provided input by reviewing item selection at two stages and providing feedback via telephone discussion. This process aimed to identify ABC-C items capturing important aspects of FXS and/or items with important HRQL impact and was iterative with statistical analysis. As a result of clinical expert input, Pearson’s correlations were calculated on the observational dataset between ABC-C items and anxiety (Anxiety, Depression and Mood Scale (ADAMS) General Anxiety Subscale, [31]), IQ, attention problems [(child and adolescent symptom inventory (CASI) [32], inattentive subscale; or adult inventory (AI) [33], inattentive subscale] and a single item global assessment of HRQL to further inform selection of items that may capture characteristics of FXS and HRQL impact not covered directly by the ABC-C.

Statistical analysis and expert input resulted in nine ABC-C items selected as key health dimensions necessary to describe the primary HRQL impacts of FXS (Table 1). All nine items are included in the revised scoring/domain structure of the ABC for FXS [16]. There are four response options for each item in the ABC-C ranging from 0—not at all a problem, to 3—severe problem. Analyses identified that the two most severe response categories (‘moderately serious’ and ‘severe’) were endorsed infrequently. As a result, these response categories were combined into a single response (‘moderately serious/severe problem’), a decision that was supported by the clinical experts.

**Table 1:** ABC-C items selected for inclusion in FXS health states, grouped by ABC-C for FXS Domain [16]

ABC-C for FXS domains [16]	ABC-C item selected for inclusion in FXS health states	
Irritability	ABC-C:4	Aggressive to other children or adults (verbally or physically)
	ABC-C:36	Mood changes quickly
Socially unresponsive/lethargic	ABC-C:58^a^	Shows few social reactions to others
Stereotypy	ABC-C:35	Repetitive hand, body or head movements
Hyperactivity	ABC-C:13	Impulsive (acts without thinking)
	ABC-C:15^b^	Restless, unable to sit still
	ABC-C:44^b^	Being easily distractible
Social avoidance	ABC-C:30^a^	Isolates him/herself from other children or adults.
Inappropriate speech	ABC-C:22	Repetitive speech

^a^Items ABC-C: 58 and ABC-C: 30 combined as a single dimension in the FXS health states

^b^Items ABC-C: 15 and ABC-C: 44 combined as a single dimension in the FXS health states

**Fig. 1:**
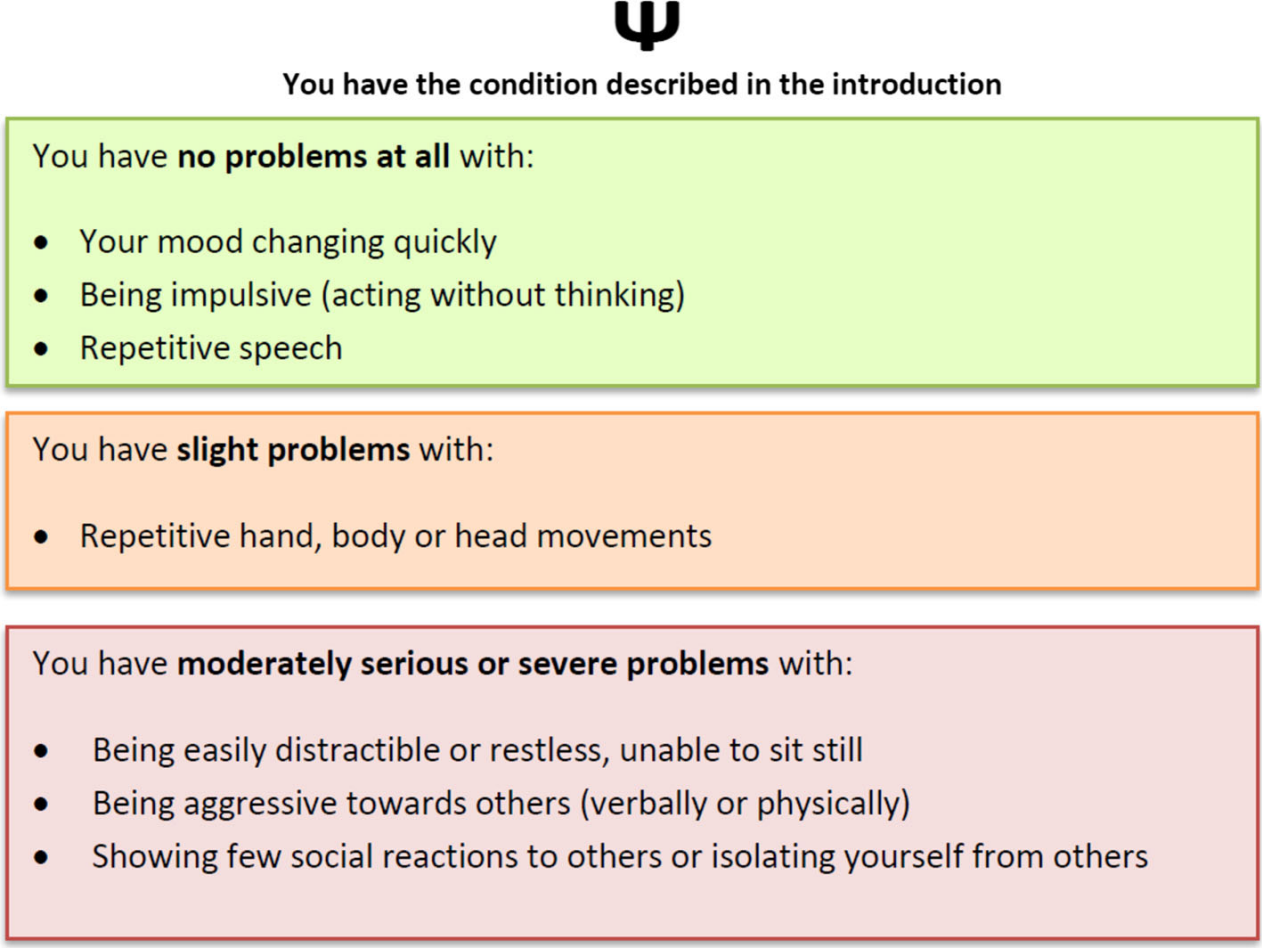
Example FXS health state

Introductory text was used to provide background information regarding FXS for study participants. This was piloted along with example health state descriptions with five members of the UK general public in cognitive debrief interviews. Following piloting, the heath state descriptions were further simplified to seven dimensions, combining items where pilot participants struggled to imagine or rate states, feeling it was illogical to experience no problems on one, but serious problems on another. These were two items related to social avoidance (ABC-C:58 showing few social reactions to other children or adults; ABC-C:30 isolating yourself from other children or adults) and two items related to hyperactivity (ABC-C:44 being easily distractible; ABC-C:15 restless, unable to sit still). Figure 1 shows an example FXS health state description derived from the seven-dimension, three-level ABC-C health state classification system and demonstrates how ABC-C item content was combined with impact levels to describe level of impact by dimension.

### Health state valuation

The seven-dimension, three-level classification system gives rise to a possible 2,187 health states. A subset of 18 health states were generated for valuation using a fractional factorial orthogonal design based on a published array (http://www2.research.att.com/~njas/oadir/oa.18.7.3.2.ugly.txt). Best (‘no problems at all’ on all seven dimensions) and worst (‘moderately serious or severe problems’ on all seven dimensions) health states were also included. The health states were randomly allocated into two sets to reduce responder burden. Each set contained the best and worst health states plus nine of the orthogonally generated health states.

After providing informed consent, general public participants were randomly allocated to one of the sets of health states. Participants read the introductory text before familiarising themselves with the health states by rating them on a 0–100 visual analogue scale (100 representing best possible health, zero representing worst possible health). Each participant was then asked to value the heath states against full health (being in the best possible state of health and not having the condition described in the introductory text). Participants valued each health state using a conventional TTO approach: participants were asked to choose whether they would prefer (A) living in generic full health for a period of time, varying from 0 to 10 years and then dying (without experiencing any time in the FXS health state), or (B) living in the health state for 10 years (remaining in that health state without improving) and then dying. The amount of time in full health in Life A was systematically changed by the interviewer until the participant indicated that they were indifferent between option A and option B. To allow for the possibility of health states being valued as worse than dead, an additional lead-time TTO (LT-TTO) valuation procedure was used for any health state where in TTO valuation a participant chose Life A when the number of years in full health was zero [indicating that the participant would prefer to die than live in the health state (B)]. In the LT-TTO procedure, participants chose whether they would prefer: (A) living for a period of time in full health, varying from 0 to 10 years and then dying (without experiencing any time in the FXS health state), or (B) living for 10 years in full health, followed by 10 years in the health state (remaining in that health state without improving) and then dying. The amount of time in life (A) was changed until the participant indicated that they are indifferent between option A and option B. As a sense check, at the end of any interview where LT-TTO had been used, participants were asked whether they were aware that they had valued the relevant state(s) as worse than dead. The combined TTO and LT-TTO valuation procedures used here were adapted from methods used for recent EQ-5D-5L valuation work [34]. In the current study, the valuation task was facilitated by a double-sided TTO/LT-TTO board.

### Sample

Three hundred and forty-nine participants, aged ≥18 years, were recruited from the UK general population using convenience sampling approaches (e.g. local advertising, word of mouth/snowballing, volunteers who had participated in previous research). Recruitment and interviews were conducted in six geographical areas of the UK. Recruitment aimed for a sample broadly representative of the socio-demographic profile of the UK general population. Socio-demographic data were collected using a background form.

### Analysis

Valuation data from the two sets of health states were combined for analysis. Regression models were fitted to the TTO/LT-TTO data (hereafter referred to as TTO data). The models are specified with level 1 or 2 of each dimension (‘slight’ and ‘moderately serious/severe’) represented by a dummy variable and ‘no problem’ the baseline reference level. These models were used to predict values for all health states defined by the classification system using the following additive regression equation:

1 $$U_{ij} = g(\beta^{{\prime }} {\mathbf{x}}_{i} ) + \varepsilon_{ij}$$

where *U* represents TTO, *i* = 1,2…*n* represents individual health states, *j* = 1,2…*m* represents respondents, *g* is a function specifying the appropriate form, **X**_***i***_ is a vector of binary dummy variables for each level *l* of dimension *d* of the descriptive system where the best level of each dimension represents the baseline for that dimension, and *εij* is an error term, whose properties depend on the assumptions of the model.

A variety of regression models were fitted to individual level data and to mean health state values: ordinary least squares (OLS), random-effects models using maximum likelihood estimation (MLE) and mean model of one mean value per state.

Performance of the regression models was assessed using the number of significant and non-significant coefficients, the consistency of the coefficients with the descriptive system, root mean squared error (RMSE) at the individual level and mean absolute error (MAE) at the state level. The Akaike information criterion (AIC) and Bayesian information criterion (BIC) were also examined. Predicted values, observed values and errors by health states were plotted and examined for patterns. The final choice of model was based on a combination of consistency and predictive performance.

## Results

### Sample characteristics

Overall, the sample characteristics were similar to census and reference data (Table 2). However, the sample was slightly younger (mean 35.8 years vs. mean England and Wales age 38.6 years [35]), included more women (58.2 % vs. 50.8 % [36]) and were more educated (54.2 % completed university vs. 27 % of the UK population have a degree-level qualification [36]).

**Table 2:** Socio-demographic sample characteristics [*n* = 349]

Age [years]	Mean [standard deviation]	35.82 [14.22]
	Median [range]	31.00 [18–83]
Gender *n* [%]^a^	Male	144 [41.3]
	Female	203 [58.2]
Quality of life (EQ-5D-3L utility)	Mean [standard deviation]	0.95 [0.09]
Ethnicity *n* [%]	White	323 [92.6]
	Mixed or multiple ethnic groups	11 [3.1]
	Asian or Asian British	10 [2.9]
	Black or Black British	1 [0.3]
	Any other ethnic group	4 [1.1]
Employment status *n* [%]	Employed full or part-time	244 [69.9]
	Student	45 [12.9]
	Seeking work/unemployed	16 [4.6]
	Retired	20 [5.7]
	Stay at home	9 [2.6]
	Other	15 [4.3]
Highest level of education *n* [%]	No formal qualifications	11 [3.1]
	Left school at 16	27 [7.7]
	Left school at 18	44 [12.6]
	Technical/vocational	61 [17.5]
	Completed university	189 [54.2]
	Other	17 [4.9]
Ever experienced a serious illness *n* [%]	Yes, in yourself	34 [9.7]
	Yes, in your family	207 [59.3]
	Yes, in caring for others	87 [24.9]

^a^Missing data *n* = 2

### Observed values for health states

The mean utility for the best health state was 0.92 [standard deviation (SD) 0.14, 95 % confidence intervals (CIs) 0.90, 0.93]. The mean utility for the worst health state was 0.16 (SD 0.47, 95 % CIs 0.11, 0.21). Mean values for all other health states fell within this range (Table 3).

**Table 3:** Observed values for health state descriptions

Health state description		Mean	Standard deviation	Minimum	Maximum	95 % Confidence intervals		Percentage of participants who valued state <0
Classification^a^								
1	2 2 2 2 2 2 2 [Worst]	0.16	0.47	−1.00	0.98	0.11	0.21	15.8
2	2 1 2 2 2 2 0	0.41	0.36	−1.00	0.98	0.36	0.47	4.0
3	2 2 1 1 2 2 2	0.43	0.40	−1.00	1.00	0.37	0.49	5.2
4	0 2 2 0 0 2 1	0.50	0.34	−1.00	0.98	0.45	0.55	2.9
5	0 0 2 0 2 1 2	0.51	0.32	−1.00	0.98	0.46	0.56	2.3
6	2 0 2 2 1 0 1	0.51	0.39	−1.00	0.98	0.45	0.56	4.6
7	1 1 2 1 0 0 2	0.53	0.36	−1.00	0.98	0.48	0.58	4.0
8	1 2 2 1 1 1 0	0.53	0.35	−1.00	0.98	0.48	0.58	4.0
9	1 0 0 2 0 2 2	0.65	0.27	−1.00	0.98	0.61	0.69	1.1
10	0 1 1 2 1 1 2	0.67	0.26	−1.00	0.98	0.63	0.70	0.6
11	1 0 1 0 1 2 0	0.67	0.24	−0.55	0.98	0.64	0.71	0.6
12	0 1 0 1 1 2 1	0.69	0.25	−0.73	1.00	0.65	0.72	1.1
13	2 0 1 1 0 1 1	0.69	0.26	−1.00	0.98	0.65	0.73	1.1
14	1 2 0 2 2 1 1	0.70	0.23	0.08	0.98	0.67	0.73	0
15	2 2 0 0 1 0 2	0.70	0.26	−1.00	0.98	0.67	0.74	0.6
16	1 1 1 0 2 0 1	0.71	0.25	−1.00	0.98	0.68	0.75	0.6
17	0 2 1 2 0 0 0	0.75	0.18	0.18	0.98	0.72	0.78	0
18	2 1 0 0 0 1 0	0.79	0.18	0.00	0.98	0.76	0.81	0
19	0 0 0 1 2 0 0	0.84	0.16	0.28	1.00	0.82	0.87	0
20	0 0 0 0 0 0 0 [Best]	0.92	0.14	0.23	1.00	0.90	0.93	0

^a^Classification: health state description by level of impairment on ABC-C items that relate to the seven health state dimensions: zero [no problems], one [slight problems] or two [moderately serious or severe]. For example, health state two [2122220] describes moderately serious or severe problems on dimensions one and three to six, slight problems on dimension two and no problems on dimension seven

See Table 4 for dimensions and corresponding ABC-C items and Fig. 1 for how health states were described for valuation

Sample size: health state descriptions 1 and 20, *n* = 349; 3, 4, 6, 7, 10, 11, 14, 18 and 19, *n* = 174; 2, 5, 8, 9, 12, 13, 15, 16 and 17, *n* = 175

### Regression analysis results

All models produced parameter coefficients indicating decrements in utility consistent with the descriptive system (i.e. all coefficients were positive, Table 4). The number of significant parameter coefficients in each model shows the importance of different dimensions. The OLS mean model produced fewest significant coefficients (Model 3, Table 4). Individual level models produced marginally lower MAEs. The absolute level of error of 0.018 compares well with previous studies using TTO to value condition-specific scales [37, 38]. None of the models show evidence of logical inconsistencies in the parameter values.

**Table 4:** Regression model estimations of weights for dimension levels

Health state dimension	ABC-C item	Variables	[1]	[2]	[3]
			OLS	RE [MLE]	Mean OLS
1	ABC-C:36	Mood1	0.025	0.025*	0.025
	Mood changes quickly	Mood2	0.083**	0.083**	0.082*
2	ABC-C:44, ABC-C:15	Distractible1	0.009	0.009	0.010
	Easily distractible or restless, unable to sit still	Distractible2	0.054**	0.054**	0.055
3	ABC-C:4	Aggressive1	0.065**	0.065**	0.067
	Aggressive towards others [verbally and physically]	Aggressive2	0.239**	0.239**	0.239**
4	ABC-C:13	Impulsive1	0.026*	0.026**	0.028
	Being impulsive, [acting without thinking]	Impulsive2	0.048**	0.048**	0.049
5	ABC-C:22	Speech1	0.022	0.022*	0.023
	Repetitive speech	Speech2	0.060**	0.059**	0.060
6	ABC-C:58 ABC-C:30	Social1	0.025	0.025*	0.026
	Shows few social reactions to others and isolating yourself from others	Social2	0.129**	0.129**	0.129**
7	ABC-C:35	Movements1	0.028*	0.028**	0.030
	Repetitive hand, body or head movements	Movements2	0.098**	0.098**	0.099*
		Constant	0.079**	0.079**	0.075
		Observations	3,430	3,430	20
		R-squared^a^	0.267		0.974
		Inconsistencies	0	0	0
		Sig. coefficients	14	14	5
		MAE	0.018	0.018	0.020
		AIC	1,444	228	-56
		BIC	1,536	332	-41

*OLS* ordinary least squares, *RE [MLE]* random effects maximum likelihood estimation, *MAE* mean absolute error, *AIC* Akaike information criterion, *BIC* Bayesian information criterion

** *p* < 0.01, * p < 0.05

^a^Due to the method of estimation, the RE [MLE] models do not produce an R-square

**Fig. 2:**
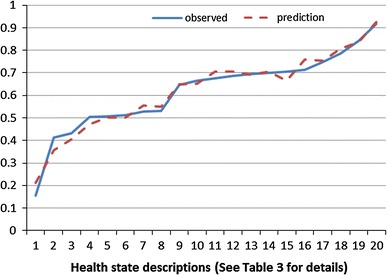
Observed and predicted health state values from the RE (MLE) model

The RE (MLE) model (Model 2, Table 4) performed slightly better than other models and is also preferred due to its ability to accommodate the repeated measures aspect of the data. Values estimated by the RE (MLE) model closely fit observed mean values for health states with little evidence for any systematic pattern to the errors produced (Fig. 2). The range in mean health state values predicted by the RE (MLE) model, 0.92 (best) to 0.21 (worst), is similar to the observed range.

### ABC-C utility index (ABC-UI)

Using the constant and parameter coefficients from the RE (MLE) model (Model 2, Table 4), a utility score is calculated by subtracting from full health (i.e. 1) the constant term and the decrement associated with the level on each dimension. The following algorithm shows how to estimate an ABC-UI score from patient-level ABC-C data:

$$\begin{aligned} U & = 1 - {\text{ Constant}}\left[ {0.079} \right] \\ & \quad -ABC36\left[ {0 = 0,1 = 0.025,2\;{\text{or}}\;3 = 0.083} \right] \\ & \quad - {\text{MAX}}\left[ {ABC15,ABC44} \right]\left[ {0 = 0,1 = 0.009,2\;{\text{or}}\;3 = 0.054} \right] \\ & \quad -ABC4\left[ {0 = 0,1 = 0.065,2\;{\text{or}}\;3 = 0.239} \right] \\ & \quad -ABC13\left[ {0 = 0,1 = 0.026,2\;{\text{or}}\;3 = 0.048} \right] \\ & \quad -ABC22\left[ {0 = 0,1 = 0.022,2\;{\text{or}}\;3 = 0.059} \right] \\ & \quad - MAX\left[ {ABC30,ABC58} \right]\left[ {0 = 0,1 = 0.025,2\;{\text{or}}\;3 = 0.129} \right] \\ & \quad - ABC35\left[ {0 = 0,1 = 0.028,2\;{\text{or}}\;3 = 0.098} \right] \\ \end{aligned}$$

where *U* represents the utility value, *ABC36* represents the score on ABC-C:36 (Mood changes quickly) and similarly *ABC4* (ABC-C:4 score, aggressive to other children or adults (verbally or physically)); *ABC13* (ABC-C:13 score, impulsive (acts without thinking); *ABC22* (ABC-C:22 score, repetitive speech); *ABC35* (ABC-C:35 score, repetitive hand, body or head movements). *MAX (ABC15, ABC44)* represents the highest score out of ABC-C:15 (restless, unable to sit still) and ABC-C:44 (being easily distractible), and similarly, *MAX (ABC30, ABC58)* represents the highest score out of ABC-C:30 (isolates him/herself from other children or adults) and ABC-C:58 (shows few social reactions to others).

## Discussion

This study was designed to develop a utility index for the ABC-C, an established outcome measure commonly used in the assessment of FXS. This utility index allows the estimation of values at the individual patient level within the range of 0.92–0.21, reflecting substantial perceived HRQL burden of problems.

The utility index was developed using relatively standard methodology where a subset of items from a validated psychometric outcome measure are used to describe health states for societal valuation. Without reducing the number of items, the valuation task would be impossibly complex and reduction of the complexity of an existing psychometric instrument is an approach common to the development of other utility indexes [28, 29]. However, the ABC-C is unusual in the extent to which the measure had to be reduced. The original instrument included 58 items [11], which meant that 80–90 % of original items had to be discarded to achieve a health state classification system of sufficient simplicity for valuation. This presented a significant challenge for retaining the validity and scope of the ABC-C.

To address this challenge, the item reduction process was guided by a number of different sources of evidence, including advanced statistical methods and expert review. Care was taken to identify items capturing a range of severity in aspects of FXS that affect males and females, children and adults, are related to cognitive and emotional characteristics of FXS and have wider impact for individuals’ HRQL. However, it should be noted that the item selection process was for the purpose of developing a utility index for HRQL and cannot be considered to reflect the entire ABC-C measure or aspects of FXS not captured by the ABC-C. As a result, although the ABC-UI reported here draws on items across the ABC-C domains [11, 16], it is designed to complement the profile of validated domain scores derived from the ABC-C and cannot be considered a proxy for a total ABC-C score, which is not considered an appropriate or valid summary score to calculate [39, 40].

In the FXS treatment context, there are several reasons why a preference-weighted adaptation of the ABC-C may better estimate utilities than an existing generic preference-weighted measure such as EQ-5D [21]. For example, the proxy-rated version of EQ-5D could have been used for assessing health status of adults and the proxy-rated EQ-5D-Y [41] could have been used with children and young people. However, the ABC-C is specific to the problems people with FXS experience, comprising conceptually very different items to those in the EQ-5D and thus has the potential to better assess the impact of disease and treatment. Secondly, the ABC-C was developed and validated as a proxy-rated measure, whereas the EQ-5D was only adapted for proxy use and the evidence to support its validity as a proxy-rated measure is limited. Indeed, there is evidence that the proxy-rated EQ-5D has poor reliability when assessing more subjective elements of health status (such as anxiety/depression and pain/discomfort) [42]. The ABC-UI offers reduced measurement burden to future clinical trials in FXS with the possibility of using a single outcome measure, as well as the potential to estimate utility values from existing ABC-C FXS datasets.

One notable aspect of the study design is the use of a combined TTO and lead-time TTO (LT-TTO) valuation method. The conventional TTO approach only allows states to be valued as better than dead, requiring the investigator to use a substantially different valuation task for states worse than dead [43]. LT-TTO offers a simpler method which is compatible with conventional TTO [44]. In the present study, the combined TTO and LT-TTO approach appears to have worked well. It was well understood by participants, and there were no obvious patterns of systematic bias in the results. Valuations by some participants in the present study that indicated a belief that certain health states were worse than being dead, were supported by interview field notes confirming that these participants understood the implications of their valuation, e.g. participants were concerned about caregiver burden and potential institutionalisation if behaviour was considered sufficiently severe.

A further methodological issue is the fact that FXS affects both children and adults. The valuation of child health states leads to some points for debate. Should adult participants value states knowing that they describe children or should they be asked to assume they are adult states? Should valuation of such states be restricted to parents because they have greater insight into the needs of children? Should the views or values of children themselves be sought? In this study, adult members of the general public were asked to value health states by imagining they are in the health state, i.e. as if they are an adult patient. This approach was taken for a number of reasons. Firstly, the ABC-C is used with children and adults and is not just a paediatric measure. Secondly, asking people to imagine that they are a child with symptoms of FXS raises concerns about introduction of bias in the data. It is not clear that people would be willing to trade years of a child’s life (even hypothetically) in order to improve their HRQL and it becomes unclear what the participant would be valuing in such an exercise. There is relatively little research that has properly addressed these issues, and it is clear that this is needed [45].

The present study has the following limitations which should be considered. It is unclear how the ABC-UI performs compared with other utility measures. This may well be important for decision-making in a reimbursement setting. One important issue is that the ABC-C is primarily a measure of behavioural problems rather than a measure focused on capturing HRQL. The QALY concept explicitly reflects survival and HRQL, and therefore, it could be argued that the ABC-UI has limitations when used to estimate QALYs. It could equally be argued that because of the nature of FXS, people’s HRQL is in large part determined by their behavioural and functional problems. However, in the valuation exercise, the respondents were provided with little or no information regarding aspects of HRQL not covered in the descriptive system, such as physical functioning. This could potentially be interpreted in different ways by respondents. This is perhaps a general limitation in the use of condition-specific measures to estimate utilities. Condition-specific measures offer the advantage of including potentially specific and sensitive items to assess the burden of a disease, but at the same time, may miss important elements of HRQL that are not affected in that disease. While it should be noted that identifying items with important HRQL impact for people with FXS was a key aim of the clinical expert review and item selection/health state development process for the ABC-UI, this does not change the fact that the ABC-UI does not specifically include aspects of HRQL commonly included in generic instruments such as physical functioning (e.g. mobility), emotional status (e.g. anxiety/depression), self-care and usual activities.

A related concern is the possibility that the focus of the ABC-C on behavioural problems rather than generic HRQL domains may have given rise to health states containing impacts which members of the general public may find hard to understand or value. In the current study, introductory text was developed with clinical expert review and input to provide background information that would help the participants’ understanding of the health states, but without naming the FXS condition. This was piloted alongside example health states, with specific probing in pilot interviews for problematic terms and difficulties general public participants may have imagining the health states described. Another potential limitation of this method is the reliance on proxy assessment for the completion of the ABC-C. However, given the nature of FXS, this remains the only realistic option for data collection.

In addition to behavioural problems, FXS is also characterised by anxiety and attention problems [3, 5], which the ABC-C is not specifically designed to capture. While this may limit the ability of the ABC-UI to reflect HRQL impact associated with less behaviourally expressed impacts, measures of anxiety and attention have been found to be highly associated with behavioural problems captured by the ABC-C in FXS, which suggest these characteristics co-occur with and may drive problematic behaviour observed [30]. Specific associations between ABC-C items and measures of non-behavioural FXS characteristics, including anxiety and attention problems, were also considered in developing the ABC-UI. Item selection was informed by the ABC for FXS social avoidance subscale [16]. However, it is possible that the behavioural focus of ABC-C socially unresponsive/social avoidance items may not characterise the specific social difficulties experienced by FXS patients, who typically seek out but struggle to cope with social situations rather than avoid or are disinterested in social interaction.

It should be noted that this work was conducted in the UK and that additional validation work may need to be conducted in other geographical areas. The UK general public sample was a convenience sample designed to approximate the general population. However, the sample differed slightly from UK general population norms. Regression models run with additional socio-demographic variables resulted in a number of the lower levels of health state dimensions becoming non-significant and showed significant effects of gender, employment and pain/discomfort (on EQ-5D). This suggests that the small differences in the sample from the general population may have had some influence on the results. However, the sizes of the parameter coefficients for the health state dimensions were very similar in the models that also included socio-demographic variables to those reported in Table 4.

Other limitations include the decision during health state development to merge the two most severe ABC-C health states. This was made on the grounds that these were infrequently endorsed in FXS, an observation that was confirmed by expert clinical input. However, it is possible that the resulting ABC-UI may lack sensitivity or demonstrate floor effects when applied to ABC-C scores from more severe FXS cases. The study also used an orthogonal design and the resulting ABC-UI assumes a linear additive functional form. This is not ideal given likely interactions among participants’ preferences across the seven health state dimensions. However, this is a common limitation of most preference-based measures, generic or condition-specific. Where attempts have been made to address non-additivity, these have not allowed for interactions between specific dimensions, but have instead assumed a constant impact that is either additive or multiplicative. To model specific interactions between dimensions requires a far larger sample size.

In conclusion, the ABC-UI appears to be able to report a wide range of utility values from patient-level FXS ABC-C data. This allows estimation of FXS HRQL impact for economic evaluation using an established instrument commonly adopted as a primary outcome measure in FXS clinical trials. However, development of a utility index from a lengthy proxy-rated measure of behaviour raises both conceptual and methodological challenges, along with questions over what the index captures and whether this is an appropriate basis from which to estimate QALYs.
